# An alternative *D. melanogaster* 7SK snRNP

**DOI:** 10.1186/s12860-021-00381-7

**Published:** 2021-08-31

**Authors:** Duy Nguyen, Nicolas Buisine, Olivier Fayol, Annemieke A. Michels, Olivier Bensaude, David H. Price, Patricia Uguen

**Affiliations:** 1 Université Paris-Saclay, INSERM, CNRS, Interactions cellulaires et physiopathologie hépatique, Bât.440, 91405 Orsay, France; 2 MNHN, UMR CNRS 7221, 75231 Paris, France; 3 IBENS Paris, UMR CNRS 8197; UA INSERM 1024, 75005 Paris, France; 4 Department of Biochemistry, University of Iowa, Iowa City, IA 52242 USA; 5 Present address: Université Paris-Saclay, CNRS, INSERM, Institut Curie, Intégrité du Génome, ARN et cancer, Bât. 110, 91401 Orsay cedex, France

**Keywords:** 7SK snRNA, Drosophila, P-TEFb, Long non-coding RNA

## Abstract

**Background:**

The 7SK small nuclear RNA (snRNA) found in most metazoans is a key regulator of P-TEFb which in turn regulates RNA polymerase II elongation. Although its primary sequence varies in protostomes, its secondary structure and function are conserved across evolutionary distant taxa.

**Results:**

Here, we describe a novel ncRNA sharing many features characteristic of 7SK RNAs, in *D. melanogaster*. We examined the structure of the corresponding gene and determined the expression profiles of the encoded RNA, called snRNA:7SK:94F, during development. It is probably produced from the transcription of a lncRNA which is processed into a mature snRNA. We also addressed its biological function and we show that, like dm7SK, this alternative 7SK interacts in vivo with the different partners of the P-TEFb complex, i.e. HEXIM, LARP7 and Cyclin T. This novel RNA is widely expressed across tissues*.*

**Conclusion:**

We propose that two distinct 7SK genes might contribute to the formation of the 7SK snRNP complex in *D. melanogaster*.

**Supplementary Information:**

The online version contains supplementary material available at 10.1186/s12860-021-00381-7.

## Background

Transcriptional elongation by RNA polymerase II (RNA Pol II) is regulated by the positive transcription elongation factor b (P-TEFb) composed of Cyclin T1 and cyclin-dependent kinase 9 (CDK9) [1]. P-TEFb is required for the release of RNA Pol II, which is paused at proximal promoter, into productive elongation. This is accomplished by the phosphorylation of both the 5,6-dichloro-1-β-D-ribofuranosylbenzimidazole (DRB) sensitivity-inducing factor (DSIF) and the Negative Elongation Factor-E (NELF-E) [2], but also by the phosphorylation of serine 2 residues on the C-terminal domain of the large subunit of RNA Pol II [3–5]. The activity of P-TEFb is regulated by the reversible association of the kinase with the 7SK snRNP [6, 7]. P-TEFb is maintained in an inactive state in the 7SK snRNP by a direct interaction with the RNA-bound, hexamethylene bisacetamide-induced proteins (HEXIM1/2) [8, 9]. 7SK is a 332-nt long non-coding RNA (lncRNA) transcribed by RNA Pol III [10–12]. In the nucleus, the 7SK snRNA is constitutively associated with Methylphosphate Capping Enzyme (MePCE; BCDIN3 homolog in *Drosophila*) which methylates its 5′ end [13], and with La-Related protein 7 (LARP7) which binds to the 3′ end [14–17]. These two factors contribute to the stability of the RNA [18–20].

The genes encoding 7SK are found in all vertebrates and the sequence of the snRNA is highly conserved in mammals, whereas basal vertebrates only exhibit poorly conserved sequences (e.g. ~ 68% similarities in lamprey) [21]. Nevertheless, their 5′ and 3′ stem-loop structures remain evolutionarily conserved, both structurally and functionally, probably because they physically interact with HEXIM, P-TEFb, MePCE and LARP7. In 2008, Gruber and colleagues [22, 23] discovered 7SK snRNA-like sequences in the genome of several protostomes (arthropods, mollusks, and annelids). Further analyses of more basal protostomes have uncovered a 7SK snRNA candidate in *Caenorhabditis* species [24], although this is still debated as it could be a homologue of the U8 snoRNA [25]. A structural analysis of the already known 7SK snRNAs has shown several highly conserved motifs in bilaterians. Thus, M1, M3 and M8 motifs may form the core structure and are closed together to stabilize the entire structure of the RNA in a “closed” structural model [24, 26]. A recent and broader analysis of the structure of invertebrate 7SK RNAs defined an additional conserved inner stem-loop structure [27]. Overall, 7SK sequences are poorly conserved among protostomes, which explains why BLAST-based searches on mammalian sequences as bait have been mostly unsuccessful. We have previously identified and characterized the 7SK snRNP containing the 444-nts 7SK RNA, P-TEFb, HEXIM, LARP7, and MePCE in *Drosophila* [28]. This indicates that the 7SK snRNA pathway is conserved in metazoans.

In this work, we identified a novel non-coding snRNA in *Drosophila* genome with a bioinformatic analysis based on the structure of the *Drosophila* RNA Pol III-specific promoters [29]. We argue that this RNA is probably not transcribed by RNA Pol III but is more likely to be formed after the cleavage of an lncRNA precursor transcribed by RNA Pol II. We document its expression levels during the life cycle*,* embryogenesis and organogenesis of *Drosophila*. We also demonstrate that this snRNA physically interacts with dmHEXIM, and co-immunoprecipitates with dmP-TEFb. We propose that this novel snRNA named snRNA:7SK:94F is a *Drosophila*-specific alternative 7SK snRNA. This is the first description of two distinct 7SK gene products which could participate in the formation of the P-TEFb snRNP complex.

## Results

### A novel snRNA in *D. melanogaster*

**Fig. 1:**
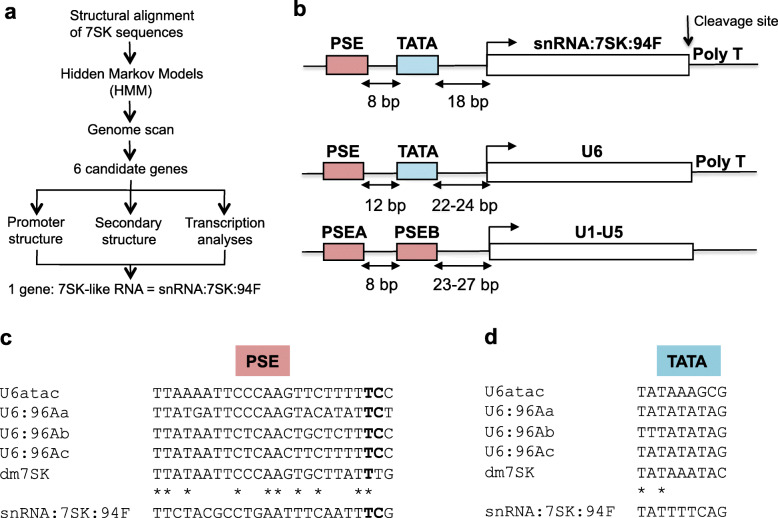
Structure of snRNA:7SK:94F gene in *D. melanogaster*. **a** Bioinformatic workflow to scan the *D. melanogaster* genome for analogues of the 7SK snRNA. **b** snRNA:7SK:94F gene structure compared to RNA polymerase III (U6 snRNA) and RNA polymerase II (U1 to U5 snRNA)-type genes. PSE: Proximal Sequence Element; PSEA and PSEB: Proximal Sequence Element type A and type B, respectively. **c** Sequence comparison of the PSE of dmU6 genes with snRNA:7SK:94F and dm7SK genes. The conserved nucleotides TC responsible for the RNA polymerase specificity are in bold characters. **d** Sequence comparison of the TATA box of dmU6 genes with snRNA:7SK:94F and dm7SK genes. The conserved nucleotides between all dmU6 genes, dm7SK gene and snRNA:7SK:94F gene are indicated by stars

The primary sequence of the 7SK snRNA is poorly conserved over large evolutionary distances, which limits the sensitivity of searches based on sequence similarities. Indeed, a BLAST search [30] failed to detect 7SK related sequences in *Drosophila* genome. Instead, we derived a profile Hidden Markov Model based on a structural alignment of chordates 7SK sequences (see Materials and Methods and Fig. 1a).

In total, six loci showed significant similarities to the 5′ or the 3′ end of the model, but only a single one of them showed > 100 bp similarity with both the 5′ and the 3′ of the model, suggesting that it corresponds to a 7SK-like RNA gene. The other loci (chr4:1169608..1169632; chrX:4501439..4501471; chr2R:12354215..12354269; chr2L:17785962..17785988; chr3L:3230335..3230369) were spurious hits arising from bioinformatic noise and were not studied further. First, we identified the Transcriptional Start Site (TSS) of this gene by 5′ RACE PCR (see Materials and Methods), which is an adenine located at 3R:23432869 (Flybase release FB2018_06) [31] (Additional file 1 Fig. A1). If the 3′ end of the gene is defined by a double track of 4 and 6 thymidines at 3R:23433272, which serves as the termination signal for RNA Pol III transcription, the putative gene would be 404 bp long (Additional file 1 Fig. A1). It is located in the 94F3 cytological band, in the second intron of the CG4374-B transcript, which encodes a protein containing a zinc finger domain of unknown function (http://flybase.org). Then we analysed the upstream region of this gene by comparison to the well-known organization of the promoters of *Drosophila* snRNA genes [29, 32, 33]. In *D. melanogaster*, the promoters of RNA Pol II (U1 to U5) and RNA Pol III (U6) snRNA genes contain two elements: an upstream Proximal Sequence Element (PSE), and a PSEB (for RNA Pol II snRNA genes) [34] or a TATA box (for U6 snRNA gene) [35]. The upstream sequences display some similarities to snRNA promoter structure: a PSE is separated by 8 bp from a TATA box, which is located 18 bp upstream of the TSS (Fig. 1b). Of note, the PSE of 7SK-like RNA contains a TC dinucleotide at position 19 and 20, typically associated with snRNA transcription by RNA Pol III in *Drosophila* (Fig. 1c). The upstream region of this locus presents only slight similarities with a TATA box although it contains a G at its 3′ end, as in insects (Fig. 1d) [29]. The internal sequence of the 7SK-like RNA gene displays 5 tracks of 4 to 5 thymidines, mainly in the first hundred nts, which are efficient RNA Pol III transcription termination signals in 75 to 95% of genes [36]. This implies that this gene, despite its Pol III-like promoter structure, is probably not transcribed by RNA Pol III, in contrast to 7SK snRNA genes found in other organisms.

**Fig. 2:**
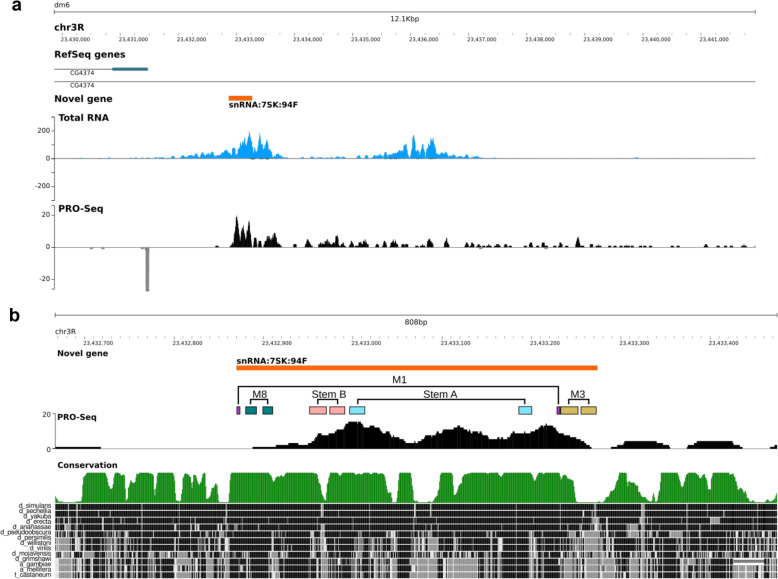
Genomic landscape surrounding of the snRNA:7SK:94F locus. **a** Precision Run-On Sequencing (PRO-Seq) analysis of nascent transcripts (GSE131160). PRO-seq signals on both forward and reverse strands are shown, together with RNA abundance measured by sequencing of total RNAs. Forward and reverse reads density are displayed with positive and negative values, respectively. **b** Evolutionary conservation of the snRNA:7SK:94F locus in arthropods. The comparison is based on 12 *Drosophilidae*, and 3 insects i.e. the honeybee (*A. mellifera*), the mosquito (*A. gambiae*) and the tribolium (*T. castaneum*) genomes. High sequence conservation is marked by black aligned blocks. Coloured boxes indicate the position of the different structural motifs (M1, M3 and M8) and stems (A and B). Tracks are available at UCSU Genome Browser, assembly version BDGP R5/dm3

RNA Pol II transcription profiling by nascent transcripts analysis with PRO-seq (Precision Run-On Sequencing) on 2–4 h embryos [37] revealed that this locus is strongly transcribed over a 4.7 kbp region (Fig. 2a). Processing and analysis of RNA-seq data generated from total RNAs extracted from *Drosophila* pupae further show strong evidence for accumulation of RNAs at the expected location (Fig. 2a). RNAs accumulation is highly consistent with PRO-seq signal. In addition, the peak of PRO-seq is slightly offset, between 20 and 60 nts downstream of the transcription start site, as observed for most genes [38].

This locus probably encodes a lncRNA that we named lncRNA:94F according to the current nomenclature. The 5′ end of this transcription unit corresponds precisely to the 7SK-like RNA sequence identified in the bioinformatic screen. It is likely that the lncRNA:94F RNA is a precursor transcript later processed into the short mature 7SK-like snRNA.

**Fig. 3:**
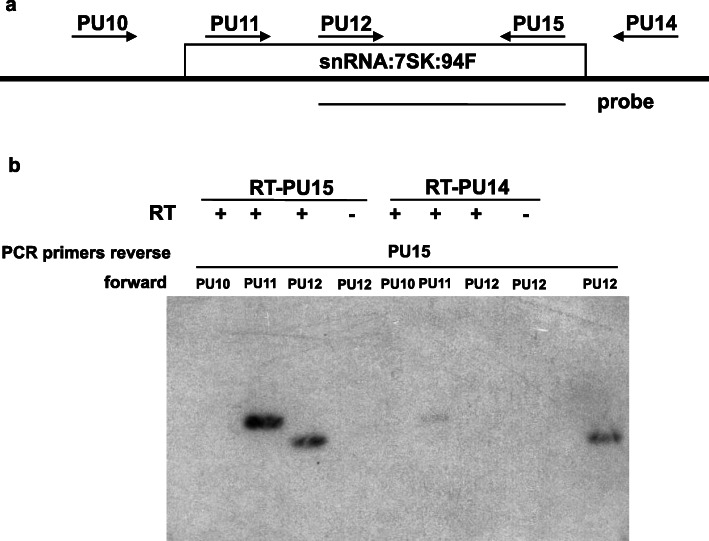
Localisation of the transcript unit of snRNA:7SK:94F gene*.* **a** Schema of the region analysed by RT-PCR followed by southern blot. The forward primers (PU14 and PU15) used to perform the reverse transcription step and PCR amplification are located on the schema. The reverse primers (PU10, PU11 and PU12) used to do PCR are also indicated. The probe used to perform hybridization on RT-PCR products is drawn under the gene structure. **b** Autoradiography of the Southern blot realized with the PU12-PU15 PCR product probe (210nts) onto different RT-PCR products which span the putative transcription unit of snRNA:7SK:94F gene. RT-PU15 indicates that the reverse transcription step has been done with PU15 primers (inside the predicted transcript region) and with PU14 primer (outside the transcript region) for the condition indicated RT-PU14. The last lane is the positive control corresponding to PCR product made with PU12-PU15 primers from the genomic DNA

The sequence of the lncRNA:94F gene is well conserved in all *Drosophilidae* species but not in other insects, particularly in the 5′ and 3′ ends (Fig. 2b). The predicted secondary structure of the 7SK-like RNA displays motifs similar to the M1, M3 and M8 motifs of the 7SK snRNA. These motifs are involved in the physical interactions within the 7SK snRNP complex [24] (Additional file 1 Fig. A2). There are also two evolutionary conserved regions, the stem A (previously called M4 and M5 motifs) [24] and the stem B [27], shared by Hexapoda. Moreover, the 5′ and 3′ ends are brought together to form the typical core structure of the 7SK RNA which is directly involved in the scaffolding of the P-TEFb inactive complex [18]. Interestingly, the different conserved structural motifs are located in the regions that are well conserved among the *Drosophilidae* (Fig. 2b). This structural analysis suggests that the 5′ end of the lncRNA:94F folds into a stable secondary structure promoting the downstream cleavage and the release of the short 7SK-like RNA, named snRNA:7SK:94F. In addition, to demarcate the processed RNA, we used RT-PCR with various forward and reverse primer sets spanning the genomic region (Additional file 1 Table A1). To increase the detection sensitivity, we carried out a southern blot on PCR-products using a radioactive probe mapping to the transcribed snRNA:7SK:94F region (Fig. 3a).

A band of the expected size is amplified only after reverse transcription (with primer PU15) followed by PCR with primers located in the predicted transcription unit (PU11 and PU12) (Fig. 3b). No stable transcript could be detected when using a reverse primer located just downstream of the putative cleavage site (PU14, Fig. 3a) or using a forward primer located upstream from the putative TSS (PU10, Fig. 3b).

### The snRNA:7SK:94F is ubiquitously expressed

**Fig. 4:**
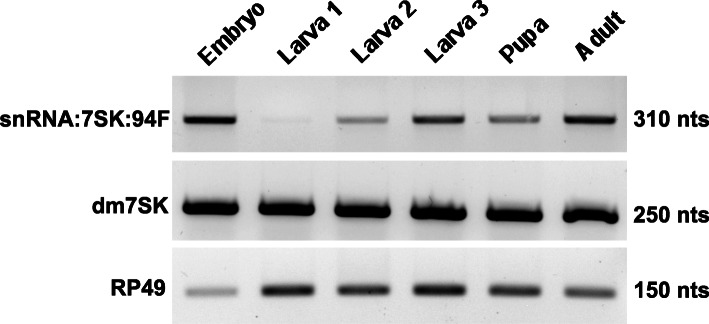
snRNA:7SK:94F is expressed during the life cycle of *D. melanogaster*. The transcription levels of the housekeeping *rp49* gene (lower panel) were used to normalize, by semi-quantitative RT-PCR, the expression levels of snRNA:7SK:94F (upper panel) and *dm7SK* (middle panel). Larva 1, 2 and 3 correspond to larval stages. The size of the RT-PCR products are indicated on the right side of the picture

We first profiled snRNA:7SK:94F expression using RT-sqPCR, from the embryonic stages until adulthood (Fig. 4).

Notably, we found snRNA:7SK:94F transcripts before zygotic activation, suggesting that it is of maternal origin in the early developmental stages. Whereas snRNA:7SK:94F transcription levels were low at the first-instar larval stage, expression strongly increased from the second-instar larva and reached peak levels at the adult stage. Of note, this is significantly different from the expression levels of the dm7SK snRNA [23], which was highly expressed at all stages during the life cycle (Fig. 4). The expression level of snRNA:7SK:94F is lower than dm7SK snRNA, between 1 to 50 times less, depending on the developmental stage (Additional file 1 Fig. A3).

We next examined in more detail the expression patterns of snRNA:7SK:94F transcripts by in situ hybridization during embryogenesis and organogenesis. snRNA:7SK:94F transcripts are ubiquitously expressed during early embryonic development (Additional file 1 Fig. A4). During the formation and extension of the germ band, snRNA:7SK:94F transcripts were strongly expressed in this embryonic zone, which was the most transcriptionally active region of the embryos. It is also expressed at later stages of embryogenesis. Likewise, during organogenesis, snRNA:7SK:94F transcripts were found in all the imaginal discs tested (eye-antenna, leg or wing discs) and larval organ brain-optic lobes (Additional file 1 Fig. A4). We also performed control experiments to ensure that these signals are specific. Indeed, no signal was detected when a sense probe against snRNA:7SK:94F was added (data not shown).

### snRNA:7SK:94F physically interacts with HEXIM

**Fig. 5:**
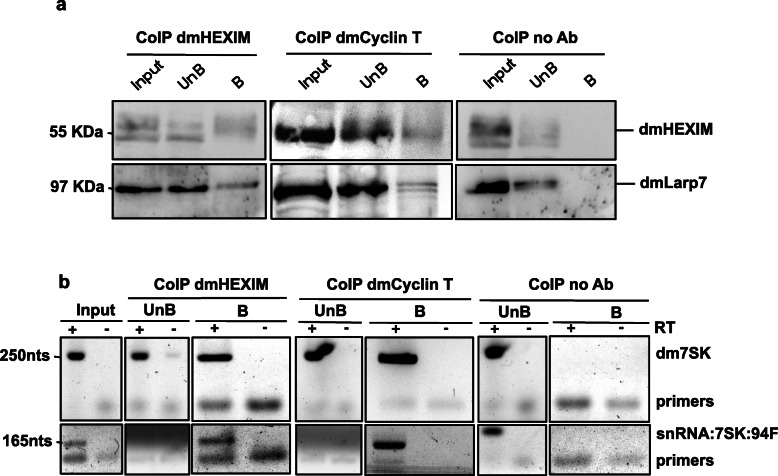
snRNA:7SK:94F interacts with P-TEFb. **a** Co-immunoprecipitation of the protein partners of HEXIM and Cyclin T from *Drosophila* adult extracts. Western blots using either HEXIM or Larp7 antibodies were performed to analyse the Input, Unbound and Bound fractions. 20% of the input and unbound fractions were loaded onto the gel. **b** RT-PCR detection of snRNA:7SK:94F (lower panel) and dm7SK (upper panel) RNAs following immunoprecipitation with dmHEXIM and dmCyclin T antibodies from *Drosophila* adult extracts. RT-PCR reactions were performed with 20% of input and unbound fractions, and with the total bound fraction. UnB: unbound fraction; B: bound fraction; RT: Reverse Transcriptase; Ab: antibody. The size of RT-PCR products or proteins are indicated on the left side of the picture

To test whether this novel snRNA shares functional properties with the 7SK RNA, we looked for physical interactions with the components of P-TEFb complexes [28]. We first performed immuno-precipitation assays with protein extracts from adult flies to explore interactions between the snRNA:7SK:94F RNA and dmHEXIM, dmLARP7 or dmCyclin T proteins. As expected, neither dmHEXIM nor dmLARP7 protein was detected in the bound fraction without antibody, whereas dmHEXIM antibodies efficiently pulled-down both dmHEXIM and dmLARP7 (Fig. 5a).

Also, dmCyclin T antibodies pulled-down dmHEXIM and dmLARP7, showing that they form a complex in vivo. Importantly, the snRNA:7SK:94F RNA co-purifies with dmHEXIM, dmLARP7 and dmCyclin T, and is completely depleted from the unbound fractions (Fig. 5b). In agreement with previous reports [28], the dm7SK RNA was also pulled-down with this complex (Fig. 5b).

**Fig. 6:**
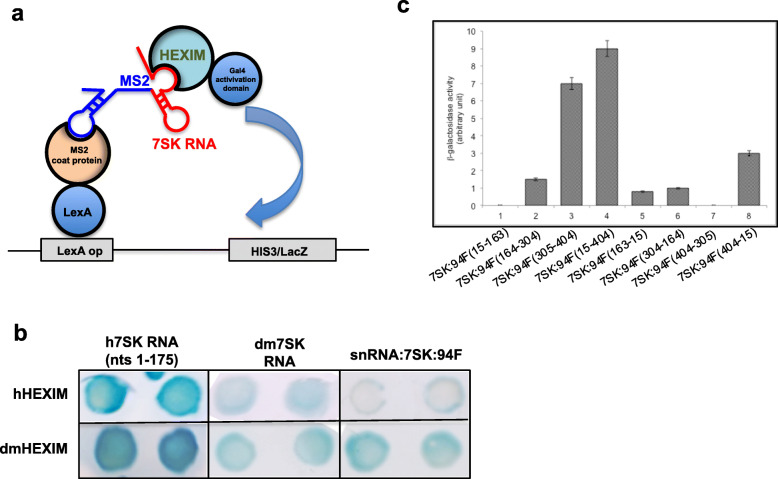
Direct interaction between snRNA:7SK:94F and dmHEXIM. **a** Principle of the yeast three-hybrid system, containing three components: the DNA-binding domain of LexA fused to MS2 coat protein, the snRNA:7SK:94F fused to the RNA MS2-binding RNA sequence, and the Gal4 activation domain fused to dmHEXIM protein. When the target hybrid RNA interacts with GAL4-HEXIM protein, the expressions of the reporter genes *lacZ* and *HIS3* were activated. **b** Qualitative analyses of the interactions between different 7SK RNAs and HEXIMs by monitoring β-galactosidase in situ. For each condition, two representative colonies are shown. h7SK RNA (1–175) is a positive control known to strongly interact with the human HEXIM ortholog (hHEXIM) [41]. **c** Quantitative analyses of the interactions between dmHEXIM and different sub-domains of snRNA:7SK:94F, either full length (nt 15–404) or shorter fragments (nt 15–163; nt 164–304; and nt 305–404) (lane 1–4). Anti-sense RNAs were used as a negative control (lane 5–8). The β-galactosidase activity was expressed in arbitrary units

We then used the yeast three-hybrid system [39, 40] to map the interaction domains of HEXIM and the snRNA:7SK:94F RNA (Fig. 6a).

Since RNAs longer than 150 to 200-nt may affect the efficiency of the assay [42], we performed the experiments with the full-length snRNA:7SK:94F RNA (nt 15–404), but also with three truncated fragments (nt 15–163; nt 164–304, and nt 305–404). The positive control is composed of human 7SK (h7SK) snRNA (nt 1–175) and human HEXIM1 (hHEXIM), which are known to interact with each other [41]. Anti-sense sequences were used as negative controls. As expected, the co-expression of MS2-h7SK (nt 1–175) and hHEXIM produced a dark blue color with X-Gal assay (Fig. 6b), indicating physical interaction between these two partners. In contrast, a light blue color was observed with MS2-h7SK (nt 1–330) and hHEXIM1, as already observed (not shown) [41]. A light blue color was detected with both dm7SK or snRNA:7SK:94F and dmHEXIM, suggesting a weak or transient (but significant) interaction (Fig. 6b). Interestingly, the h7SK RNA displayed a strong interaction with dmHEXIM indicating that hHEXIM and dmHEXIM are functionally interchangeable. By contrast, *Drosophila* snRNA:7SK:94F did not interact with hHEXIM (Fig. 6b). The activation of the reporter genes using these constructs was also confirmed by monitoring the β-galactosidase activity in liquid assays (Fig. 6c). We observed a 3-fold increase of the β-galactosidase activity when MS2-dm7SK:94F fragments (nt 15–404 or nt 305–404) were co-expressed with dmHEXIM (Fig. 6c). These observations suggest that snRNA:7SK:94F interacts with dmHEXIM mainly through its 3′ end. Thus, in *Drosophila*, this experiment confirms that dmHEXIM interacts with two distinct 7SK snRNAs.

## Discussion

In this study, we discover a novel 7SK-like snRNA in *Drosophila* genomes. We propose that it is generated after cleavage of a 4.7 kb lncRNA precursor, probably transcribed by RNA Pol II. The mature RNA displays strong structural similarities with known 7SK snRNA, and engages physical interactions with the various components of the P-TEFb complex. For these reasons, we propose that this novel processed lncRNA is an analogue of the 7SK snRNA and shares overlapping biological activities. According to the international nomenclature of ncRNA, we named the precursor, lncRNA:94F and the mature RNA, snRNA:7SK:94F.

The 7SK snRNA is highly abundant in eukaryotic cells and is well known for its crucial role in the regulation of RNA Pol II-mediated transcription. Although they have been found in the genome of many vertebrates (from humans to lampreys) [21], homologues of 7SK snRNA have been only recently described in several protostomes [22–24, 27]. Notably, the 7SK *Drosophila* homologue is able to sequester P-TEFb into a large and catalytically inactive complex contributing to RNA Pol II elongation control [28, 43], as seen in vertebrates.

7SK snRNA homologues have been found in bilaterians and follow precisely the phylogenetic distribution of HEXIM [24]. This is not surprising since the components of molecular complexes often exhibit very similar phylogenetic distribution. RNAs generally co-evolve with their respective partners and they are subject to strong structural constraints, albeit with a relaxed selection at the sequence level [44, 45]. As such, the sequence of lncRNA is often poorly conserved and distant homologues/analogues are notoriously difficult to find and many of these may have been missed in genomes. Of note, the internal part of 7SK snRNAs is less constrained by physical interactions with a partner (e.g. HEXIM, P-TEFb, LARP7 and MePCE), which certainly relaxes locally the selection pressure and may favours sequence variations. This easily explains the difference of the gene length observed in Drosophiladae (~ 400 nts) compared to Vertebrates (~ 300 nts). In addition, the evolution of lncRNA is known to occur at a fast pace and genome turnover is important in *Drosophila* [46]. There are many examples where the biological function of lcnRNA does not depend on its primary sequence but rather on its 3D structure [47]. All the known 7SK snRNA display a core structure containing M1, M3, and M8 motifs [24]. The stem A is well conserved as well, although it has not yet been linked to a function. In invertebrates, the 7SK snRNAs, together with snRNA:7SK:94F, present an additional conserved stem B structure [27]. We were able to detect this novel 7SK analogue because of the exquisite sensitivity of HMMs, but also because its 3′ end (which physically interacts with LARP7) is fairly similar to that of humans. Importantly, this novel 7SK displays all the most conserved regions found among the invertebrates 7SK RNA.

An intriguing fact is that the sequence of snRNA:7SK:94F presents several internal tracks of thymidines which act as RNA Pol III transcriptional termination signals. It is therefore highly unlikely that it is an RNA Pol III gene. An interesting hypothesis would be that the RNA Pol III promoter structure evolved over time into an RNA Pol II promoter which overcomes premature termination. Accordingly, the promoter specificity is not dictated by the TATA box but rather by the sequence of the PSE [32]. Such a functional shift would only require a few point mutations and would be very rapid over evolutionary time. The transcription unit almost certainly generates a lncRNA which might be further processed into the snRNA:7SK:94F. The folding with strong secondary structure probably helps direct cleavage to the precise site corresponding to the 3′ end of the snRNA:7SK:94F. As such, folding would be biologically relevant at two levels: for physical interactions with components of the P-TEFb complex, and for definition of the post-processing cleavage site. Processing from longer precursors is not unusual in lncRNAs, since it is already observed in the case of MALAT1 (Metastasis-associated lung adenocarcinoma transcript 1) and NEAT1 (Nuclear enriched abundant transcript 1). Indeed, these two lncRNAs are transcribed as 8 to 22.7 kb-long pre-RNAs, respectively, and then, they are processed by the RNAse P ribonucleoprotein complex into shorter lncRNAs [48].

What are the functional connections (if any) between these two 7SK genes found in *Drosophila*? Do they functionally cooperate or is their functions antagonistic or independent of each other (despite interacting with the same regulatory complex)? The fact that more of one 7SK sequence is present in a genome is not new. For example, mammalian genomes contain a single functional 7SK gene but they are populated with numerous 7SK-like retro-pseudogenes [49]. This raises the question of the possible role of those pseudogenes as molecular sponge, similarly to PTENP1 which is the pseudogene of PTEN protein [50]. Moreover, many lncRNAs function as molecular sponges for miRNA or RNA Binding Protein (RBP) to limit the abundance of their functional binding sites [51, 52]. In previous study, two 7SK gene copies were found in *C. elegans* genome, but the expression of only one could be detected by Northern blot, probably due to the very low expression level of the other. The authors suggest that they are paralogues subjected to concerted evolution because their sequence is nearly identical [23]. Likewise, expression of snRNA:7SK:94F is challenging to measure as we were unable to detect it by Northern blot and we had to use highly optimized RT-PCR protocol to detect it. Nonetheless, the case for *Drosophila* is quite different from *C. elegans*: the sequences of the dm7SK and the proposed snRNA:7SK:94F are poorly conserved and display distinct expression patterns during development. This suggests that the two genes are not fully redundant and may have specific biological functions. Also, one can note that the phylogenetic distribution of snRNA:7SK:94F is restricted to the *Drosophila* genus (Fig. 2), suggesting that this snRNA is an innovation specific to this group and may have a *Drosophila*-specific function.

Proteins can often accommodate various molecular functions. For example, Actin proteins are components of the cytoplasmic acto-myosin network, but they are also nuclear components of the chromatin remodeling complex [53]. Beta-catenin, a membrane bound protein and transcription factor, is another example [54]. This is much less common for RNA, and although several cases have been reported [55, 56], their corresponding function is often restricted to the biology of nucleic acids. For example, U1 snRNA presents different roles in regulation of genes expression [57]. In humans, the 7SK snRNA itself has different roles, and some are dependent on the P-TEFb complex [58] and some are independent [59, 60]. Therefore, given the known function of HEXIM, it is tempting to speculate that the function of the HEXIM/snRNA:7SK:94F complex may be related to transcriptional regulation. It is clear however, as stated above, that snRNA:7SK:94F and dm7SK snRNAs do not have redundant functions because snRNA:7SK:94F is differentially expressed with lower levels in the larval and pupal stages whereas dm7SK displays high and constant expression during development. Although snRNA:7SK:94F physically interacts with the components of the P-TEFb complex, it does so in a slightly different manner to dm7SK, since the GAUC stem, which is the contact interface between HEXIM and 7SK, is mutated into GAUG [61]. Mutations in this 5′-hairpin have been extensively studied and some can affect the binding efficiency to HEXIM [62]. Thus, the point mutation found in snRNA:7SK:94F may have a functional impact in vivo. An interesting hypothesis (albeit untested) would be that snRNA:7SK:94F could be a molecular sponge aimed at regulating the abundance of the proteins forming the 7SK snRNP complex.

## Materials and methods

### Identification of a 7SK-like analogue in *D. melanogaster*

Given that ncRNA 7SK sequences are poorly conserved, we used the HMMER package (www.hmmer.org) to look for signatures of 7SK sequences. To this end, a structural alignment built from the 7SK sequence of humans, mice, chicken, xenopus, fugu, tetraodon, lamprey, myxin and amphioxus was used to set up a nucleotidic multiple alignment, which was used to derive a profile HMM model with hmmbuild. The model was used to scan the *Drosophila* genome (FB2018_06 release) [31] with hmmsearch. Alignments were local for both the genome and the model.

### Processing of NGS datasets

Sequence reads were mapped on Dmel v6 genome available from UCSC website by using the bowtie short reads aligner [63] with stringent mapping parameters (−m 1 -n 1 -l 49 -p 20). Reads were clipped down to 50 bp, as this does not limit mapping efficiency and specificity but prevents issues with spliced alignments. Genome wide density profiles were computed with genomeCoverageBed from the BEDTOOLS package [64] and converted to bigwig with the wigToBigWig software, from the Jim Kent suite available at http://hgdownload.soe.ucsc.edu/admin/jksrc.zip. Visualization of reads density and the genomic environment is carried out with the JBROWSE genome browser [65]. RNA-Seq datasets (SRA reference PRJNA644503; https://www.ncbi.nlm.nih.gov/bioproject/PRJNA644503) from wild type pupae are from [66] and PRO-seq data (GEO reference GSE131160; https://www.ncbi.nlm.nih.gov/geo/query/acc.cgi?acc=GSE131160) are from Ueberschar et al. [37].

### 5′ RACE PCR, semi-quantitative RT-PCR

Ten micrograms of RNA, extracted from adult flies (Qiagen RNeasy kit), were reverse- transcribed with the SuperScript II reverse transcriptase (RT II, Invitrogen). The 5′ end of the snRNA:7SK:94F was identified by the Rapid Amplification of cDNA End (RACE) system (Invitrogen) as previously described [67], using specifically designed primers (Additional file 1 Table A1). The purified PCR products were cloned into the TA cloning vector (Invitrogen). Nine clones were screened for the presence of an insert, which was sequenced afterwards.

Semi-quantitative RT-PCR (RT-sqPCR) were carried out with RT II and hexamer oligonucleotides from Invitrogen. Series of PCRs were performed from purified cDNAs to amplify PCR fragments of snRNA:7SK:94F, dm7SK, and RP49 RNAs with corresponding primers (Additional file 1 Table A1). PCR was performed in a two-step procedure. The first step, aimed at improving specificity, an initial denaturating step during 5 mins at 94 °C was followed by 10 cycles of 30 s at 94 °C, 30 s at annealing temperature (starting at 55 °C, plus 0.5 °C per cycle) and 30 s at 72 °C. The second step, aiming to amplify the signal, corresponds to 25 cycles of 30 s at 94 °C, 30 s at 60 °C and 30 s at 72 °C. The PCR was completed by an additional incubation of 7 mins at 72 °C. The cDNAs were successively diluted 5-fold until reaching the less saturated dilution for RP49 PCR amplification. This dilution factor was estimated for each cDNA sample (from 5^6^ to 5^8^-fold). Then the relative dilutions of the cDNAs were used to estimate relative snRNA:7SK:94F and dm7SK PCR quantities.

RT-PCR were carried out as described below, using two different reverse primers (Additional file 1 Table A1) and a series of forward primers spanning the predictive transcribed region of snRNA:7SK:94F gene. In order to improve the sensitivity of the detection, a southern blot was realized on the PCR-products using a radioactive probe corresponding to the PU12-PU15 PCR-product. PCR-products were loaded onto a 1.5% agarose gel and transfered to a Hybond N+ membrane, hybridized with the probe and washed with 2X SSC containing 0,1% SDS, then washed with 0.1X SSC containing 0,1% SDS at 50 °C. Autoradiography was performed for a few minutes on XOMAT films. Probes were labeled by nick translation (Thermofisher) using [α-^32^P]dCTP.

### Secondary structure

The secondary structure of hs7SK snRNA, snRNA:7SK:94F, and dm7SK snRNA was computed with the mfold software, a predictive RNA-folding program [68]. The structure of hs7SK snRNA served as a control, since it has been extensively studied by different methods [12, 24]. Using mfold, the hs7SK snRNA is folded into a structure similar to the one determined by Wassarman and Steitz [12].

### In situ hybridization

In situ hybridizations of embryos (a mix of 0-4 h and 0-18 h aged embryos) and larval tissues were carried out with standard procedures [69] using appropriate primers (Additional file 1 Table A1). Each experiment was replicated three times on at least 30 embryos and 15 larvae, and lead to identical results. Embryos or imaginal discs were mounted on slides and captured by microscope (Zeiss, X100).

### Co-immunoprecipitation

Co-immunoprecipitations were performed using rabbit antibodies against isoform A of dmHEXIM [28], and sheep antibodies against dm Cyclin T [70]. Ten micrograms of purified antibodies were bound and crosslinked to 30 μl of G/A sepharose magnetic beads (Crosslink magnetic CoIP kit; Pierce). Then, 750 μg of protein extract from 60 adult flies were incubated overnight at 4 °C with antibodies. The unbound fraction was harvested and the bound fraction was eluted into 50 μl. Protein partners were analysed by western blot using primary antibodies dmHEXIM (1/2000) and dmLARP7 (1/2000) [28]. A Clean Blot IP buffer (Thermo-Scientific) was used as a secondary antibody at 1/2000. Immunodetection was performed with ECL (GE Healthcare) and imaged with the Fusion Fx7 system (BioRad). RNA partners from the bound or unbound fractions were extracted and analysed with specific primers (Additional file 1 Table A1).

### Yeast three-hybrid system

The yeast three hybrid system was used as previously described [39, 42]. pIIIA/MS2-derived plasmids carry the *URA3* gene and express snRNA:7SK:94F fragments fused to two MS2 sites. The pACTII-derived plasmids carry the *LEU2* gene and drive the production of the dmHEXIM isoform A (dmHEXIM A) [28] fused to the Gal4 activation domain. A dmHEXIM A PCR fragment was cloned into the *Bam*HI and *Xho*I sites of pACTII, after amplification from the pET21-HEXIMA plasmid [28] using specific primers (Additional file 1 Table A1). Full-length or truncated snRNA:7SK:94F fragments were amplified from genomic DNA of *D. melanogaster* CantonS strain (Bloomington *Drosophila* Stock Center) and cloned into the *Sma*I sites of pIIIA/MS2.1 using specific primers (Additional file 1 Table A1). Qualitative and quantitative analyses of β-galactosidase activities were performed as previously described [42].

## Supplementary Information

**Additional file 1: Fig. S1** Sequence of the snRNA:7SK:94F region. Positions of PSE and TATA box are underlined. The transcription start site is indicated over the + 1 nucleotide. The RNA sequence is in bold case and the untranscribed region is in lower case. The position and orientation of the different structural motifs i.e. M1, M3 and M8 and stems A and B are indicated by arrows onto the sequence. R: reverse orientation; F: forward orientation. **Fig. S2** Secondary structure of hs7SK snRNA, snRNA:7SK:94F and dm7SK snRNA. The structures have been defined by mfold software [62] to allow comparison. The remarkable conserved structures (M1, M3 and M8 motifs) [23], the less conserved one (stem A and B) [26] or the highly conserved sequence (GAUC) [59] are indicated on the structures. The minimum free energy, *DG*, is indicated next to the structure. **Fig. S3** Quantification of the relative expression levels of dm7SK RNA and snRNA:7SK:94F at different developmental stages. The histogramm displays the ratio of dm7SK RNA expression levels versus snRNA:7SK:94F. Expression levels normalized over RP49. Average of two to three different representative experiments. **Fig. S4** Expression patterns of snRNA:7SK:94F during embryogenesis and organogenesis. **a** Expression patterns of snRNA:7SK:94F were monitored by in situ hybridization. Embryos are oriented anterior to the left, dorsal uppermost. They are ordered by developmental stages. **b** In situ hybridization during organogenesis in eye-antenna, leg and wing imaginal discs, and brain from third-instar larvae. **Fig. S5** Uncropped image of the gel used to analyse the localisation of the transcript unit of snRNA:7SK:94F gene, as shown Fig. 3. The legend is similar to Fig. 3. **Fig. S6** Uncropped image of the gels used to analyse the expression of snRNA:7SK:94F during the life cycle of *D. melanogaster*, as shown Fig. 4. The legend is similar to Fig. 4. **Fig. S7** Uncropped image of the gels used to analyse by co-immunoprecipitation the interactions between HEXIM and cyclin T, as shown Fig. 5a. The legend is similar to Fig. 5a. **Fig. S8** Uncropped image of the gels used to analyse by co-immunoprecipitation the interactions between HEXIM, cyclin T, and dm7SK or snRNA:7SK:94F as shown Fig. 5b. The legend is similar to Fig. 5b. **Fig. S9** Uncropped image of the photographies used to analyse direct interaction between snRNA:7SK:94F and dmHEXIM by three-hybrid system, as shown Fig. 6. The legend is similar to Fig. 6. **Table S1** Name and sequence of primers used in this work.

## Data Availability

The datasets analysed during the current study are available from the SRA website under the reference PRJNA644503, and the GEO repository under the reference GSE131160.
