# Supplementary material for: An alternative D. melanogaster 7SK snRNP
Source: BMC Mol Cell Biol. 2021 Aug 31;22:43. doi: 10.1186/s12860-021-00381-7 (PMC8406779; doi:10.1186/s12860-021-00381-7)
Supplement: Supplementary file 1 — Additional file 1: Fig. S1 Sequence of the snRNA:7SK:94F region. Positions of PSE and TATA box are underlined. The transcription start site is indicated over the + 1 nucleotide. The RNA sequence is in bold case and the untranscribed region is in lower case. The position and orientation of the different structural motifs i.e. M1, M3 and M8 and stems A and B are indicated by arrows onto the sequence. R: reverse orientation; F: forward orientation. Fig. S2 Secondary structure of hs7SK snRNA, snRNA:7SK:94F and dm7SK snRNA. The structures have been defined by mfold software [62] to allow comparison. The remarkable conserved structures (M1, M3 and M8 motifs) [23], the less conserved one (stem A and B) [26] or the highly conserved sequence (GAUC) [59] are indicated on the structures. The minimum free energy, DG, is indicated next to the structure. Fig. S3 Quantification of the relative expression levels of dm7SK RNA and snRNA:7SK:94F at different developmental stages. The histogramm displays the ratio of dm7SK RNA expression levels versus snRNA:7SK:94F. Expression levels normalized over RP49. Average of two to three different representative experiments. Fig. S4 Expression patterns of snRNA:7SK:94F during embryogenesis and organogenesis. a Expression patterns of snRNA:7SK:94F were monitored by in situ hybridization. Embryos are oriented anterior to the left, dorsal uppermost. They are ordered by developmental stages. b In situ hybridization during organogenesis in eye-antenna, leg and wing imaginal discs, and brain from third-instar larvae. Fig. S5 Uncropped image of the gel used to analyse the localisation of the transcript unit of snRNA:7SK:94F gene, as shown Fig. 3. The legend is similar to Fig. 3. Fig. S6 Uncropped image of the gels used to analyse the expression of snRNA:7SK:94F during the life cycle of D. melanogaster, as shown Fig. 4. The legend is similar to Fig. 4. Fig. S7 Uncropped image of the gels used to analyse by co-immunoprecipitation the interactions betwee [file 12860_2021_381_MOESM1_ESM.docx]

**An alternative *Drosophila melanogaster* 7SK snRNP**

Duy Nguyen^a^, Nicolas Buisine^b^, Olivier Fayol^a^, Annemieke A. Michels^c^, Olivier Bensaude^c^, David H. Price^d^, and Patricia Uguen^a*1^

**Additional file**

**Additional figure A1.**


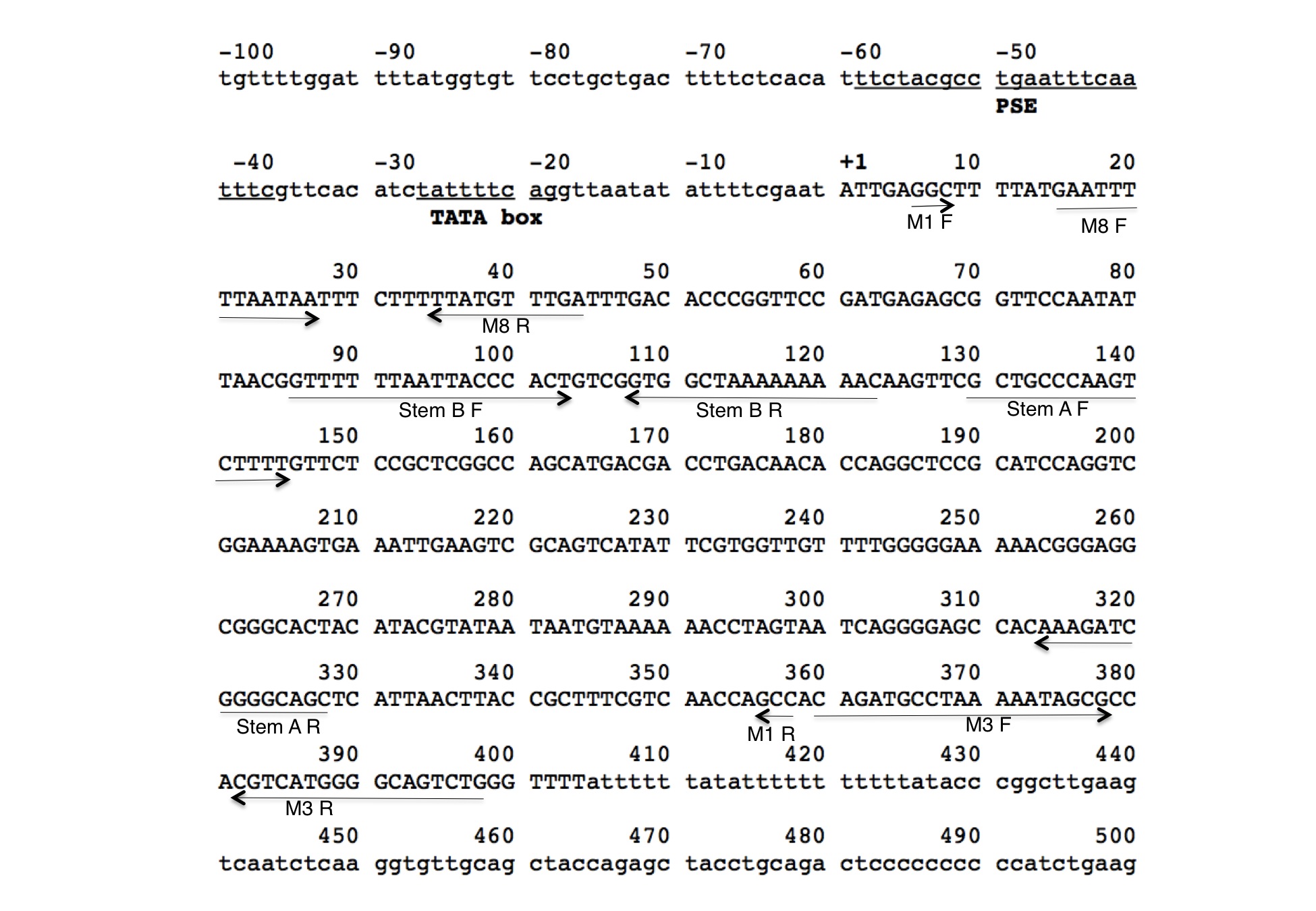


**Additional Figure A1** Sequence of the snRNA:7SK:94F region. Positions of PSE and TATA box are underlined. The transcription start site is indicated over the +1 nucleotide. The RNA sequence is in bold case and the untranscribed region is in lower case. The position and orientation of the different structural motifs *i.e*. M1, M3 and M8 and stems A and B are indicated by arrows onto the sequence. R: reverse orientation; F: forward orientation.

**Additional Figure A2.**

**
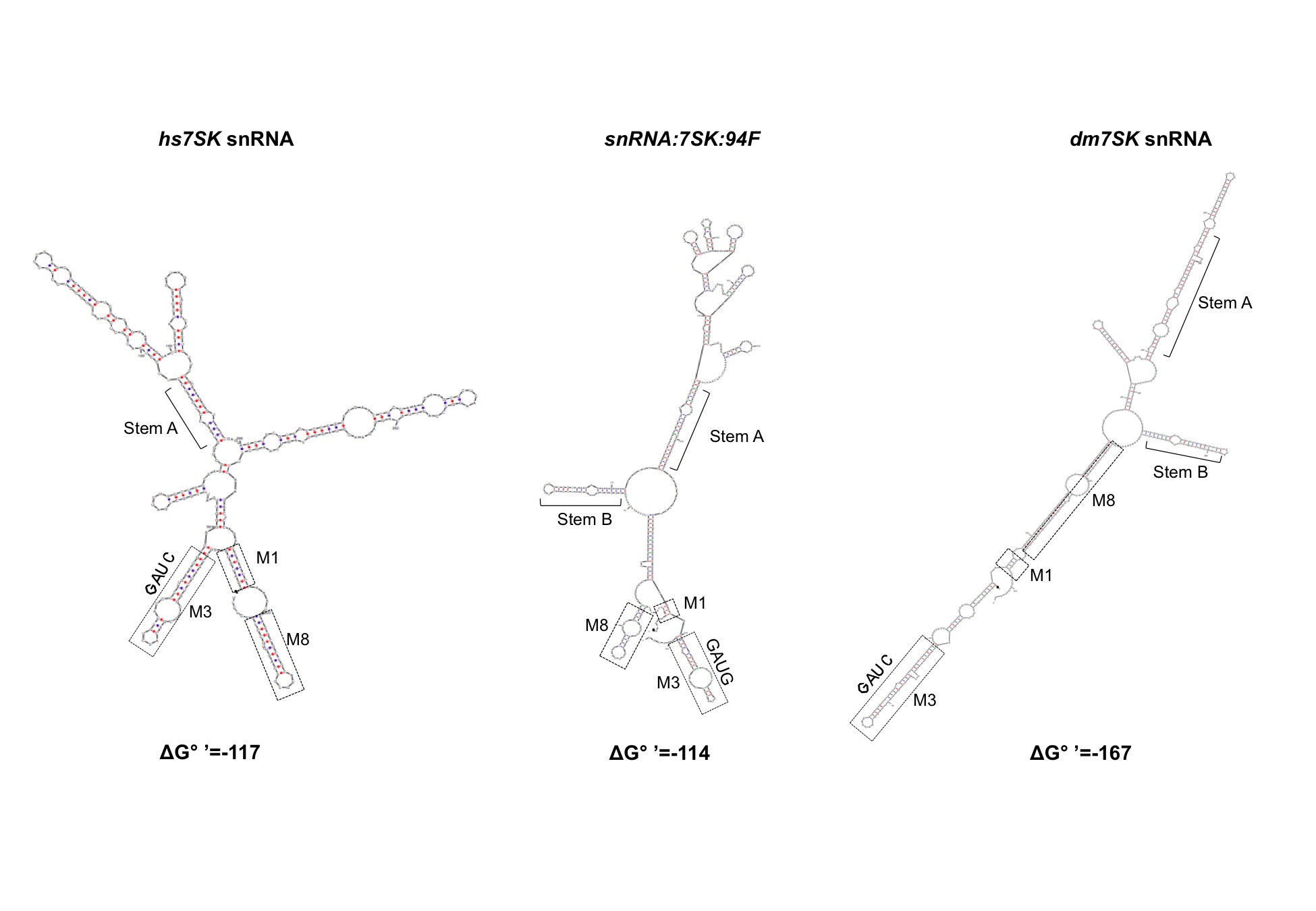
**

**Additional Figure A2** Secondary structure of hs7SK snRNA, snRNA:7SK:94F and dm7SK snRNA. The structures have been defined by mfold software [62] to allow comparison. The remarkable conserved structures (M1, M3 and M8 motifs) [23], the less conserved one (stem A and B) [26] or the highly conserved sequence (GAUC) [59] are indicated on the structures. The minimum free energy, *DG*, is indicated next to the structure.

**
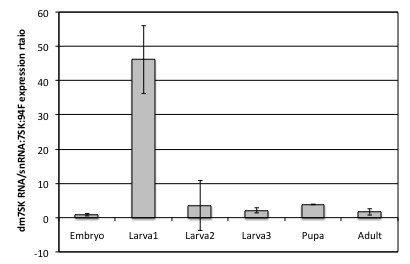
Additional Figure A3.**

**Additional Figure A3** Quantification of the relative expression levels of dm7SK RNA and snRNA:7SK :94F at different developmental stages. The histogramm displays the ratio of dm7SK RNA expression levels versus snRNA:7SK:94F. Expression levels normalized over RP49. Average of two to three different representative experiments.

**Additional Figure A4.**


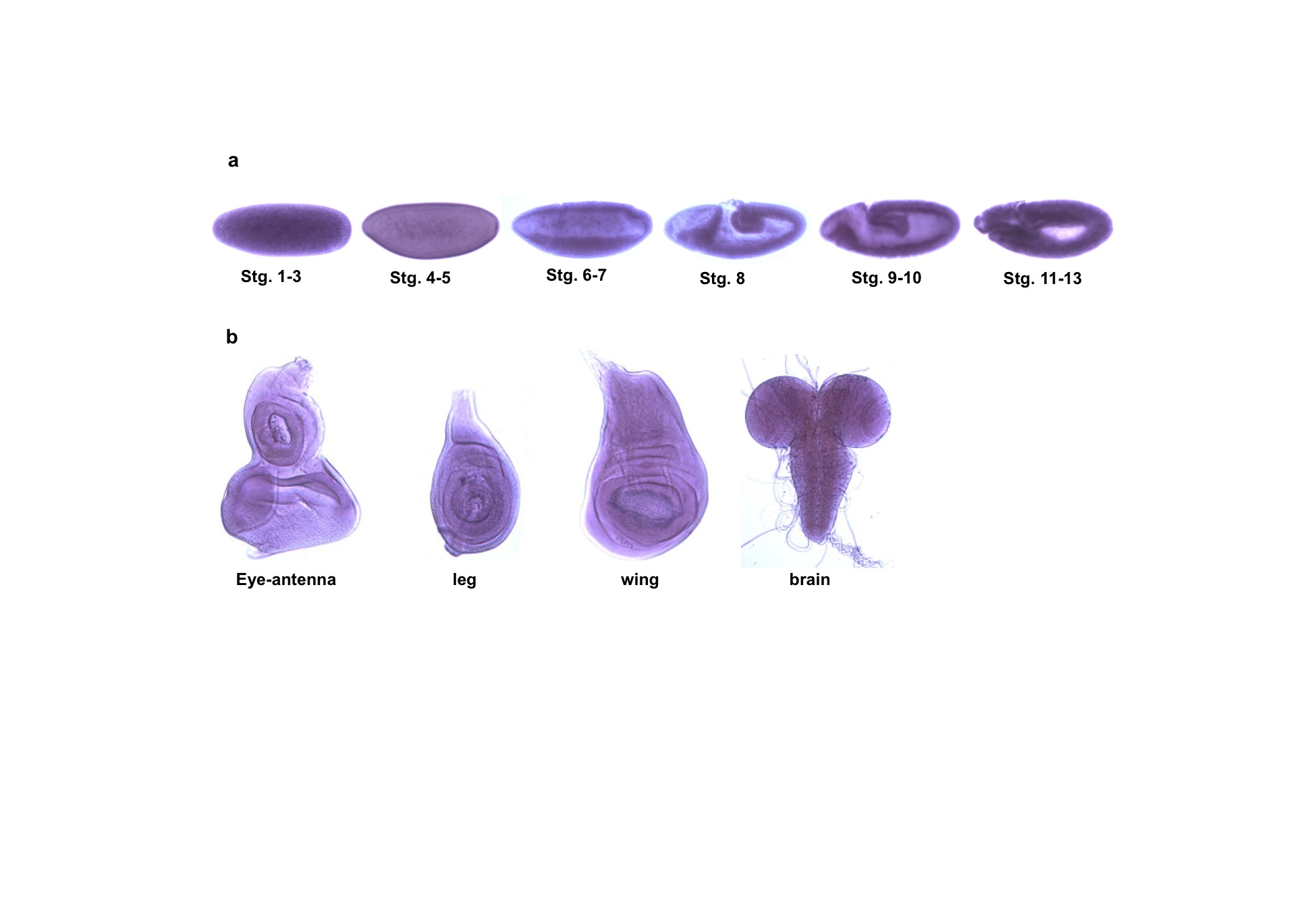


**Additional Figure A4** Expression patterns of snRNA:7SK:94F during embryogenesis and organogenesis. **a** Expression patterns of snRNA:7SK:94F were monitored by *in situ* hybridization. Embryos are oriented anterior to the left, dorsal uppermost. They are ordered by developmental stages. **b** *In situ* hybridization during organogenesis in eye-antenna, leg and wing imaginal discs, and brain from third-instar larvae.

**Additional Figure A5.**


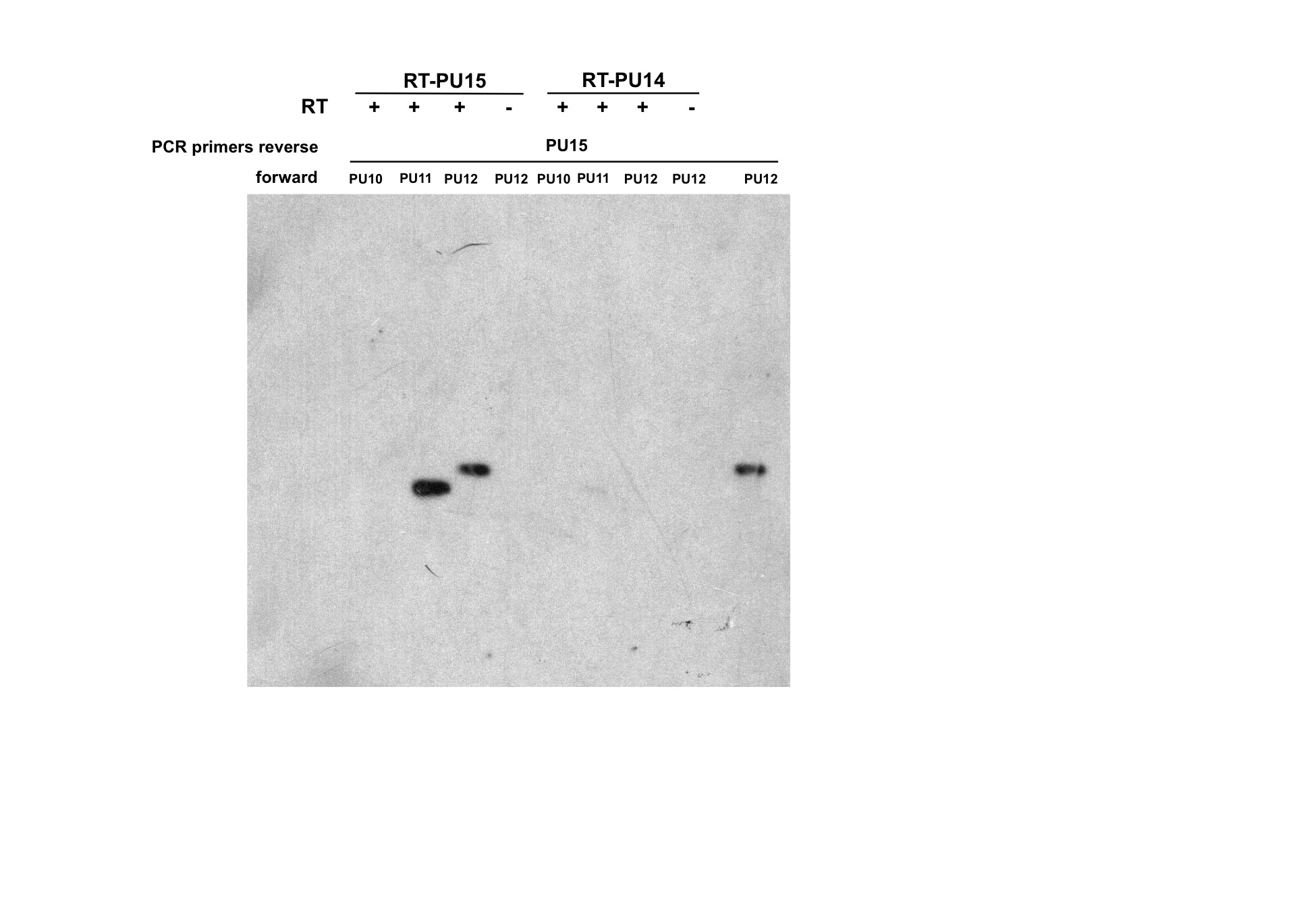


**Additional Figure A5** Uncropped image of the gel used to analyse the localisation of the transcript unit of snRNA:7SK:94F gene, as shown figure 3. The legend is similar to figure 3.

**Additional Figure A6.**


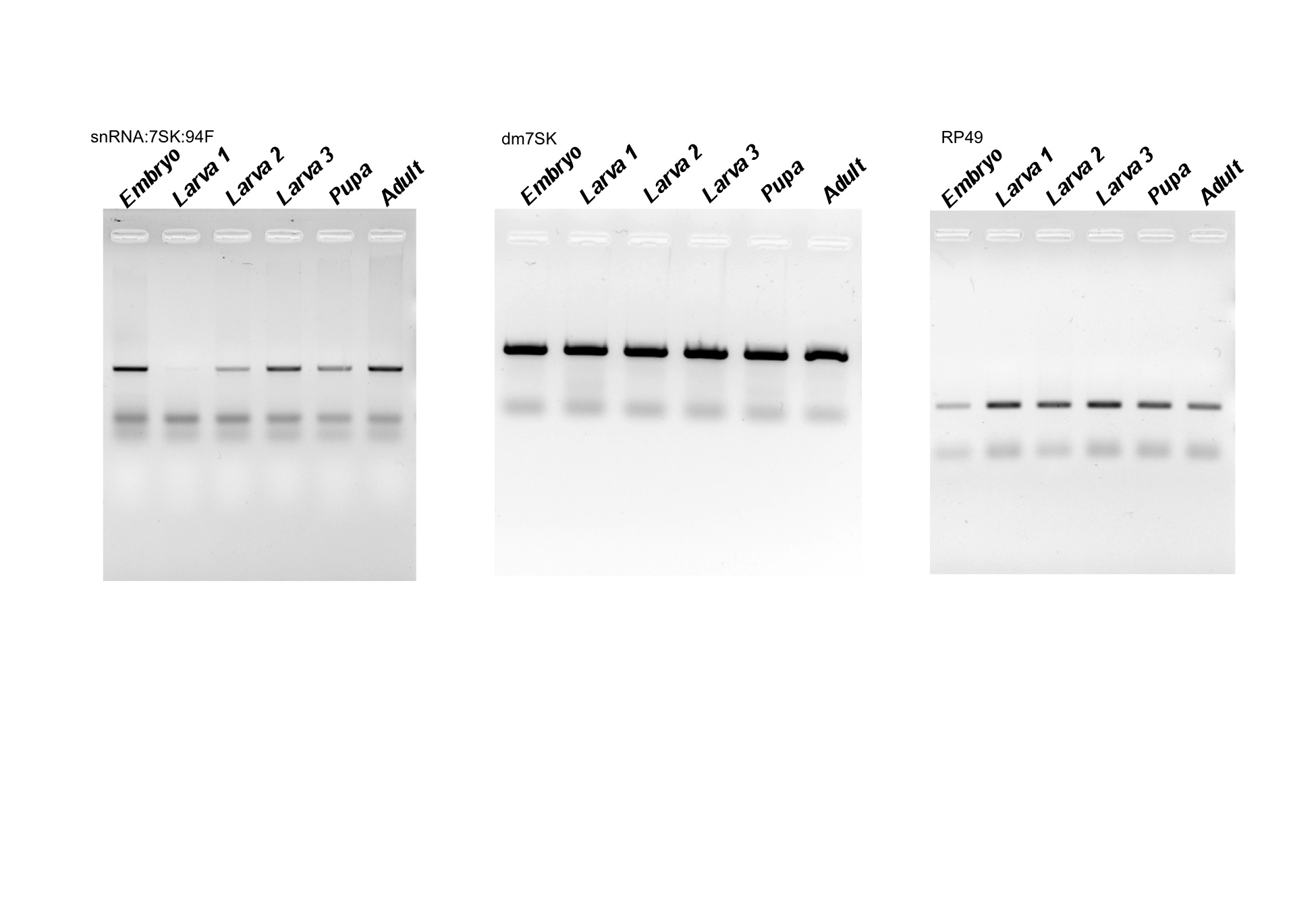


**Additional Figure A6** Uncropped image of the gels used to analyse the expression of snRNA:7SK:94F during the life cycle of *Drosophila* *melanogaster*, as shown figure 4. The legend is similar to figure 4.

**Additional Figure A7.**

**
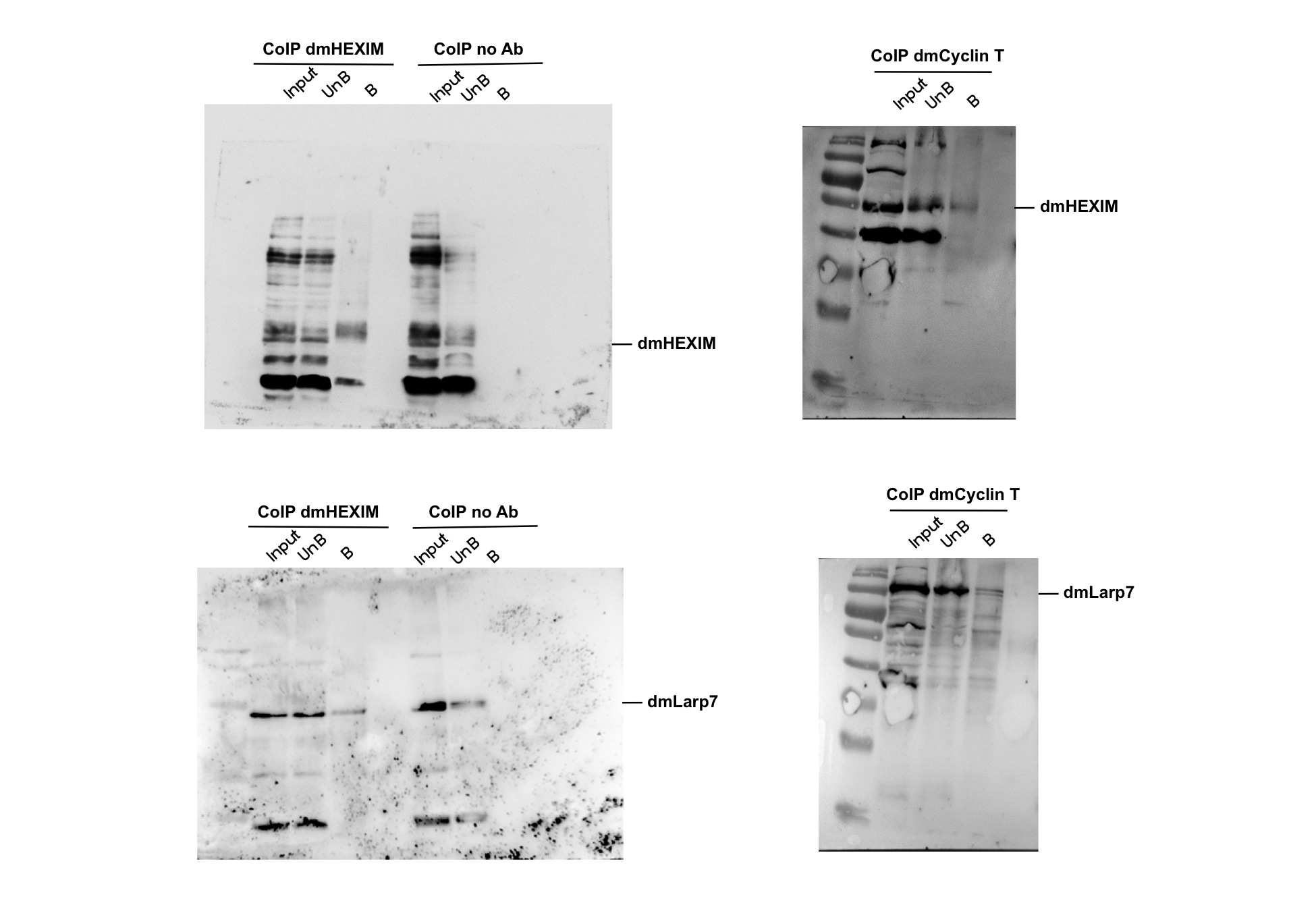
**

**Additional Figure A7** Uncropped image of the gels used to analyse by co-immunoprecipitation the interactions between HEXIM and cyclin T, as shown figure 5a. The legend is similar to figure 5a.

**Additional Figure A8.**


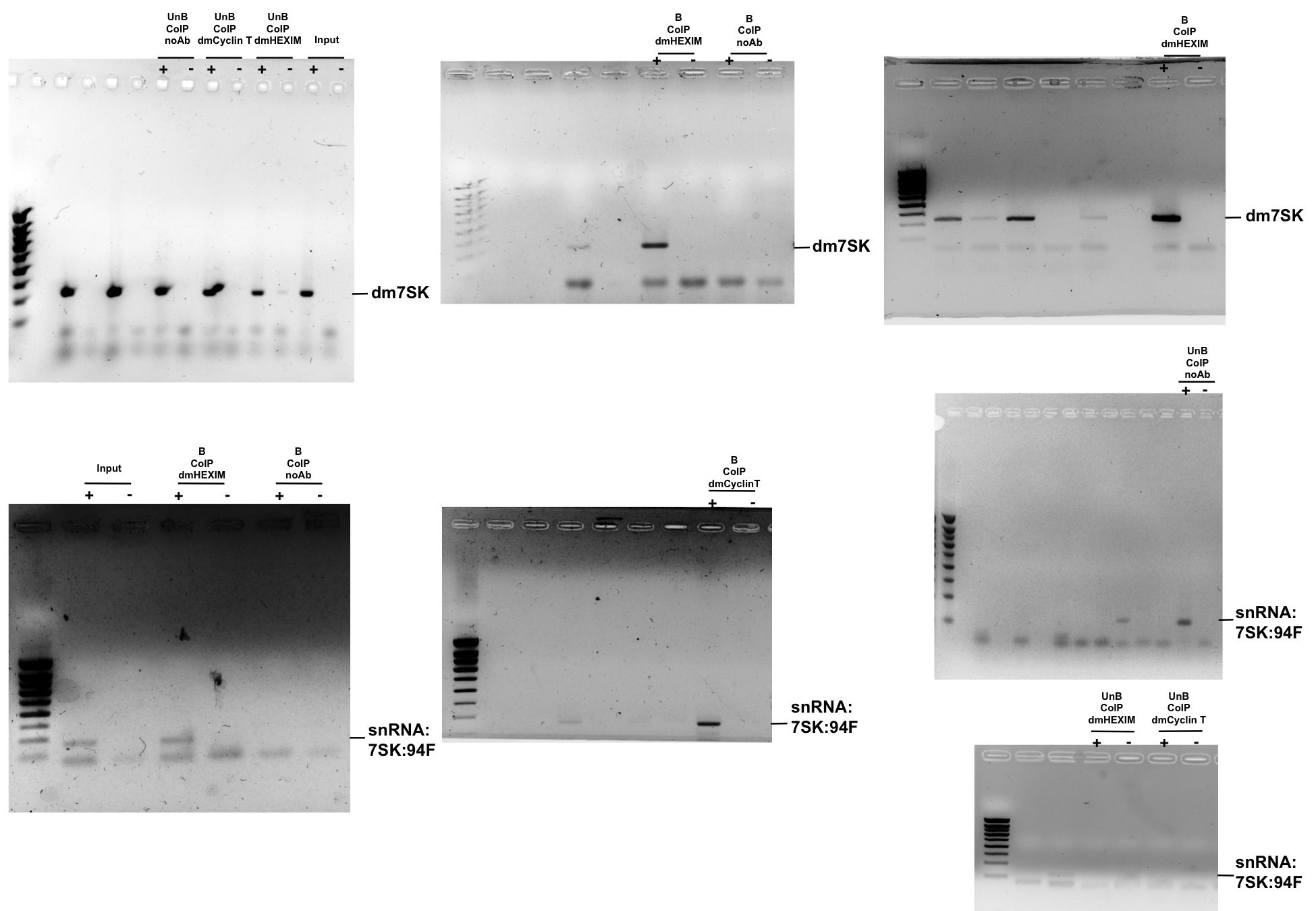


**Additional Figure A8** Uncropped image of the gels used to analyse by co-immunoprecipitation the interactions between HEXIM, cyclin T, and dm7SK or snRNA:7SK:94F as shown figure 5b. The legend is similar to figure 5b.

**Additional Figure A9.**


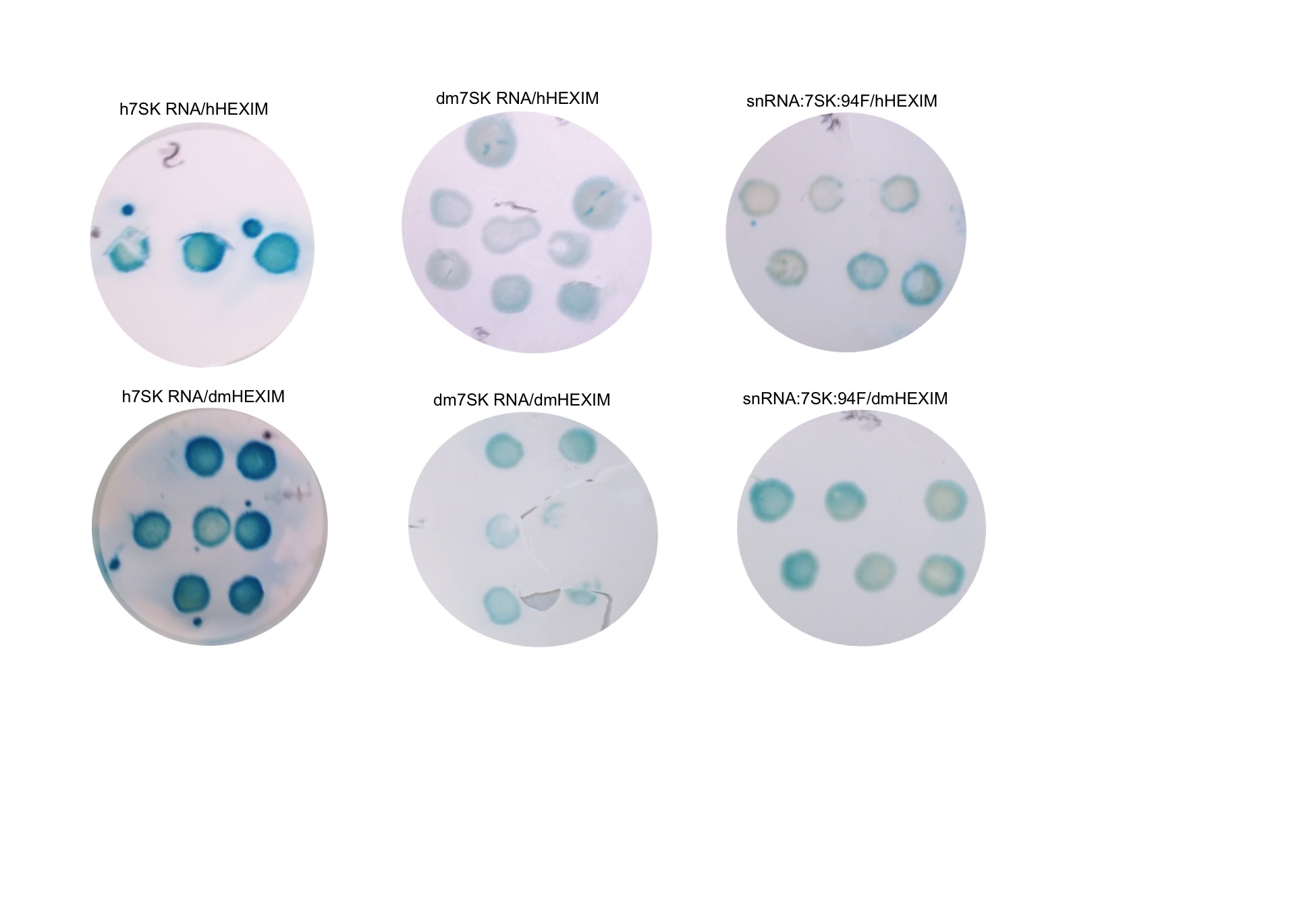


**Additional Figure A9** Uncropped image of the photographies used to analyse direct interaction between snRNA:7SK:94F and dmHEXIM by three-hybrid system, as shown figure 6. The legend is similar to figure 6.

**Additional table A1**

**Table A1** Name and sequence of primers used in this work

| **Name** | **Sequence (5’ to 3’)** | **Experiment** |
| --- | --- | --- |
| GSP1 | CGTCAACCAGCCACAGATGC | 5’ RACE (RT step) |
| GSP2 | GATCGGGGCAGCTCATTAACT | 5’ RACE (first nested PCR) |
| custom AAP | GGCCACGCGTCGACTAGTACGGGIIGGGIIGGGIIG | 5’ RACE (first nested PCR) |
| nested GSP | CCTAGTAATCAGGGGAGCCAC | 5’ RACE (second nested PCR) |
| AUAP | GGCCACGCGTCGACTAGTAC | 5’ RACE (second nested PCR) |
| PU10 forward | TTTCAATTTCGTTCACATCTAT | RT-PCR snRNA:7SK:94F |
| PU11 forward | CGGTTCCGATGAGAGCG | RT-PCR and RT-sqPCR snRNA:7SK:94F |
| PU12 forward | CTCGGCCAGCATGACGA | RT-PCR snRNA:7SK:94F |
| PU14 reverse | CTCTGGTAGCTGCAACAC | RT-PCR snRNA:7SK:94F |
| PU15 reverse | ATCTGTGGCTGGTTGACG | RT-PCR and RT-sqPCR snRNA:7SK:94F |
| PU118 forward | ACTGGATGACGACGGGTTAT | RT-sqPCR dm7SK and CoIP dm7SK |
| PU119 reverse | CCGCGTACATGGAAGTGTT | RT-sqPCR dm7SK and CoIP dm7SK |
| RP49 forward | GGCCCAAGATCGTGAAGAAG | RT-sqPCR RP49 |
| RP49 reverse | CCGATGTTGGGCATCAGATAC | RT-sqPCR RP49 |
| PU40 forward | TAATACGACTCACTATAGGAACCCAGACTGCCCCATGA | In situ snRNA:7SK:94F |
| PU41 reverse | CGGTTCCGATGAGAGCGG | In situ snRNA:7SK:94F |
| PU129 forward | GACGACCTGACAACACCA | CoIP snRNA:7SK:94F |
| PU130 reverse | TAATGAGCTGCCCCGATC | CoIP snRNA:7SK:94F |
| PU15-404 forward | ATACCCGGGAATTTTTAATAATTTCTTTTTATGTT | 3 hybrid snRNA:7SK:94F (15-404nts) |
| PU15-404 AS | AATCCCGGGAAAATAAAACCCAGACTGCCC | 3 hybrid snRNA:7SK:94F (15-404nts) |
| PU15-163 forward | ATACCCGGGAATTTTTAATAATTTCTTTTTATGTT | 3 hybrid snRNA:7SK:94F (15-163nts) |
| PU15-163 AS | TAACCCGGGCTGGCCGAGCGGAGAACA | 3 hybrid snRNA:7SK:94F (15-163nts) |
| PU164-304 forward | ATACCCGGGCATGACGACCTGACAACAC | 3 hybrid snRNA:7SK:94F (164-304nts) |
| PU164-304 AS | TTACCCGGGTGATTACTAGGTTTTTTACATTAT | 3 hybrid snRNA:7SK:94F (164-304nts) |
| PU305-404 forward | ATACCCGGGGAGCCACAAAGATCGG | 3 hybrid snRNA:7SK:94F (305-404nts) |
| PU305-404 AS | AATCCCGGGAAAATAAAACCCAGACTGCCC | 3 hybrid snRNA:7SK:94F (305-404nts) |
| PUHexim forward | TCTGAAGGATCCTGGCTGAAGCTGTAAA | 3 hybrid dmHEXIMA |
| PUHexim AS | ACGTCTCCTCGAGCTATTTGACAACTGCAT | 3 hybrid dmHEXIMA |
